# Sachi Prasad Ray-Chaudhuri: *Drosophila* genetics and mutagenesis in Indian science

**DOI:** 10.1093/mutage/geag016

**Published:** 2026-04-01

**Authors:** Rajiva Raman, Awadhesh N Jha

**Affiliations:** Cytogenetics Laboratory, Department of Zoology, Banaras Hindu University, Varanasi 221005, India; School of Biological and Marine Sciences, University of Plymouth, Plymouth, Plymouth PL4 8AA, UK

**Keywords:** radiation genetics, mutagenesis, cytogenetics, *Drosophila*, Ray-Chaudhuri

## Abstract

This article summarizes the scientific contributions of Professor Sachi Prasad Ray-Chaudhuri and his unwavering commitment to advancing research and teaching in mutagenesis and genetics in post-independence India, following his postgraduate training in the United Kingdom under the mentorship of Professor H. J. Muller. His contributions in basic mutagenesis, radiation protection, animal cytogenetics, and population genetics using a range of model and native species laid the foundation of scientific research in India in these contemporary fields. This played a pivotal role in advancing genetics and biological sciences in India at a time when scientific infrastructure and resources were limited. He also took the initiative to modernize the curriculum of biology teaching in India in line with the developments in the subject globally. He expanded these efforts by teaching as a visiting professor in different, often remote parts of the country, where he inspired the students. The seeds of his relentless effort to connect the advances in Indian science with those in the rest of the world have blossomed, and Indian science today contributes significantly to the global knowledge base. His pioneering scientific contributions and inspiring legacy continue unabated.

## Introduction

**Figure 1:**
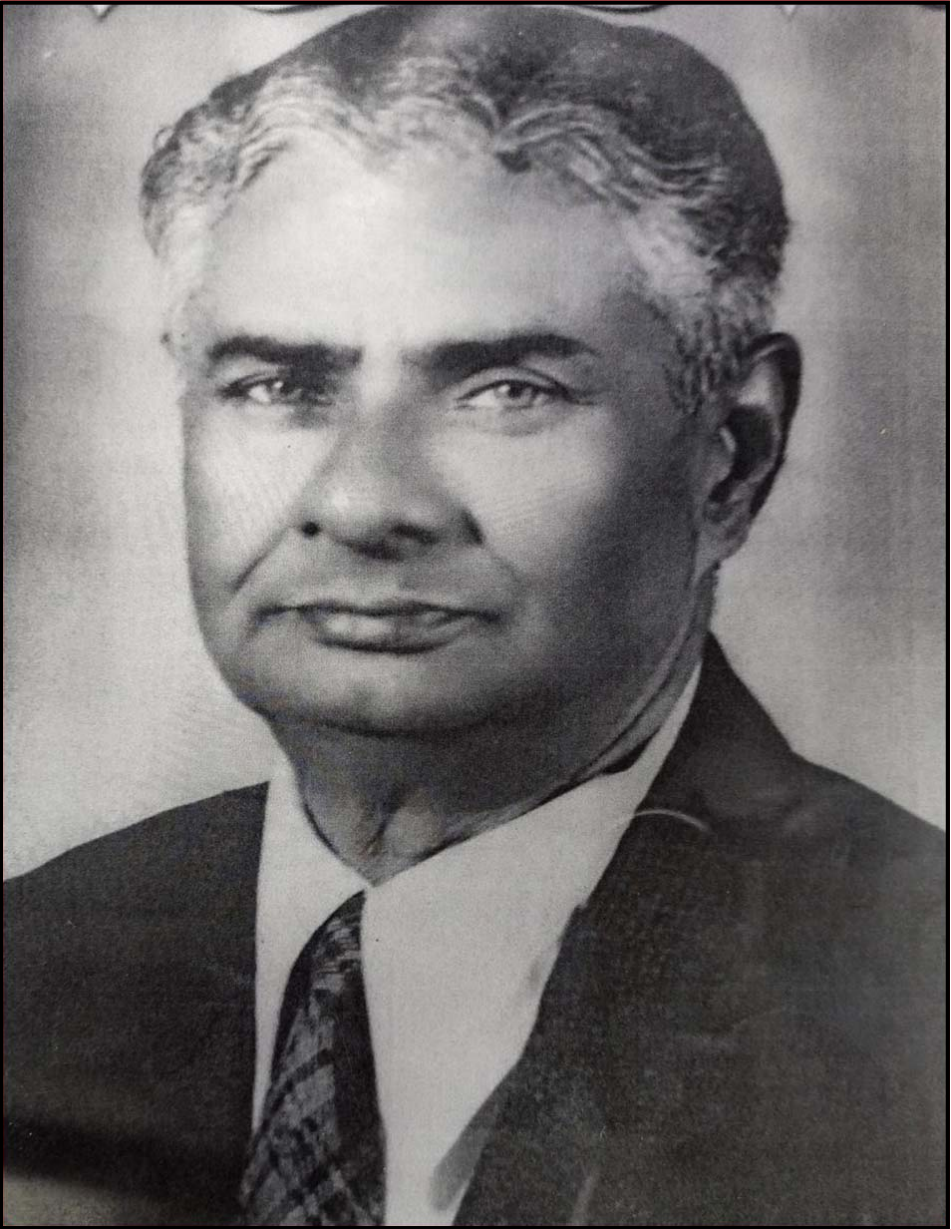
Photograph of Professor S.P. Ray-Chaudhuri taken around 1980.

The formal growth of research and teaching of Mutagenesis, Animal Genetics, and Cytogenetics in India owes its origin and expansion in a large measure to Sachi Prasad Ray-Chaudhuri (Fig. 1). How that came about is an interesting story that we will take up later in this reflective article. Ray-Chaudhuri was born on 15 September 1907 to Rama Prasad, a civil engineer attached to the Public Works Department of the Government, and Prafulla Nalini Ray-Chaudhuri in the Bagura District of Bengal (now part of Bangladesh). Since his father had a transferable job, Ray-Chaudhuri spent most of his early childhood with his paternal uncle in Bankura district of North Bengal, where he had his schooling. He later moved to Calcutta (now Kolkata) for further studies, obtaining an M.Sc. degree in Zoology from Calcutta University in 1930. In 1935, he married Nilima, daughter of a renowned geologist, Dr Hemchandra Dasgupta. They had a daughter and a son. Ray-Chaudhuri passed away on 15 February 1994 at his Calcutta residence at the age of 87.

## Initiation at the Institute of Animal Genetics, Edinburgh, UK

As a student, Ray-Chaudhuri had shown some inclination towards Genetics, which was still an obscure branch of biology in Indian Universities. Since silk was a flourishing industry in India, Ray-Chaudhuri went to the UK in 1937 to work on silkworm genetics in the Institute of Animal Genetics, Edinburgh, and registered for a Ph.D. with Prof. F.A.E. Crew, who was the Director of the Institute and an authority on silkworm genetics. Events took a different turn when H.J. Muller, renowned *Drosophila* geneticist, landed at the Institute as a fugitive from the Communist Soviet Russia in 1938. Russia was then deeply invested in the fraudulent science of T.D. Lysenko who tried to prove that nurture, not nature (Genetics), is the guiding process of growth, development, and evolution. Muller, who managed to escape from Russia and reached Edinburgh started his sojourn with a series of lectures at the Institute of Animal Genetics, that he would deliver every afternoon, elucidating various facets of genetics, focusing especially on induced mutagenesis. At the end of each lecture he would ask if anyone was interested in pursuing the questions raised in the lecture as a research problem. Ray-Chaudhuri was so thoroughly impressed by these lectures that he too decided to join him to work on fruit flies (*Drosophila)* instead of silkworms. Prof. Crew most graciously allowed him to join Muller for his Ph.D. Ray-Chaudhuri worked on the production of mutations by low-intensity radiation in *Drosophila*. Muller had made foundational contributions in genetics, specifically in the area of ionizing radiation-induced mutagenesis, which led him to receive the Nobel Prize in Physiology and Medicine in 1946. His work on X-ray-induced mutations in fruit flies, *Drosophila*, not only made it possible to collect more mutant flies but also raised concerns against the damage caused to the genetic material by ionizing radiation. Ray-Chaudhuri’s work on the effect of low intensity radiation on the induction of mutations in *Drosophila* showed that the frequency of mutations was proportional to the dose of radiation but was independent of the intensity of radiation (Radiation dosage 2000r and 400r). The mutation rate was also independent of the duration of exposure to radiation [1]. It was an extremely important finding, especially in the context of the ongoing world war, the nuclear explosions in 1945, and increasing use of radiation in day-to-day life. Ray-Chaudhuri’s work demonstrated that, at least in the fly, there was no threshold radiation in dosage as low as 400r and rate as low as 0.01r/minute. Muller referred to Ray-Chaudhuri’s work repeatedly in his Nobel Lecture [2]. This issue has drawn much attention and discussion through the years, especially with respect to human exposure.

By the time Ray-Chaudhuri received his Ph.D. degree in 1940, Europe was already embroiled in World War II, which prevented him from leaving for India immediately after completing the work. He used this period to learn techniques of chromosome preparation from P.C. Koller, a faculty member at the Institute of Hungarian origin. Koller remained a trusted friend, mentor, and later an examiner of the Ph.D. theses of a number of his students.

## Linearity no threshold (LNT) radiation effect and mutation model

Any account on Ray-Chaudhuri’s work will be incomplete without revisiting his Ph.D. work, which, as stated above, inferred that there was no threshold below which the exposure to radiation was safe. First presented in 1939 at the VII International Genetics Congress held in Edinburgh, the full paper was published in 1944 [1]; (appended as supplementary material 1). Muller, who got Nobel Prize in 1946 for his work on radiation-induced mutagenesis, referred to Ray-Chaudhuri’s work several times in his Nobel Lecture. Though done in his lab under his supervision, Muller encouraged Ray-Chaudhuri to publish it in sole authorship, crediting him for the planning and execution of the experiments all by himself.

However, more than half a century later, Edward Calabrese, an American toxicologist, wrote a barrage of articles between 2009 and 2025 discrediting the research that led to the conclusion that there was no threshold safe radiation dose. Reserving the most severe of his barbs against Muller, the Nobel Laureate, he tried to prove that the works done by Ray-Chaudhuri and later by Curt Stern and his group as faulty, incongruous, and deliberate misrepresentations of the data. In later articles, his targets included some more celebrated geneticists, viz., George Beadle, Ed Lewis (both Nobel laureates), James Neel, and others. When Calabrese wrote these articles, none of them was alive. However, Calabrese’s grossly inflated, confusing, and mostly incorrect charges have been firmly answered and rebutted through numerous articles, which deflate his claims with copious rational answers [3, 4].

In one of his articles, referring to Ray-Chaudhuri’s work, Calabrese [5] wrote ‘Muller neglected to point out key limitations of the Ray-Chaudhuri experiment [1] such as poor temperature control, changing the strain of fruit fly midway through the study, and yet still combining data of the two strains to gain statistical power without providing any justification. There was also insufficient detailing of research methods, inadequate data on quality control parameters, as well as a failure to provide information on age selection criteria for males, sex ratios of offspring, and rates of sterility and fecundity as well as data on lethal clusters, all of which are important in this type of study. The Ray-Chaudhuri study also employed a dose rate that was significantly greater than that employed by Caspari and Stern [6]. If Muller were really thinking that some type of systematic error or other experimental factor might have been overlooked, the Ray-Chaudhuri study gave more than ample reason for concern’. More innuendos, including on the person of Ray-Chaudhuri, were reserved for his other articles. Since these articles were written more than a decade after Ray-Chaudhuri’s death, and 70 years after the publication of those results, there was no possibility of a response on Ray-Chaudhuri’s behalf.

For the benefit of readers, we append here Ray-Chaudhuri’s original paper (Supplementary Material 1), which was the subject of Calabreses’s criticism. As expected, it has all the hallmarks of a first paper coming out of a student’s thesis. Using a rather simple language, he described methods elaborately, also pointing out the difficulties and limitations of the experimental strategies. He repeated the experiments eight times and presented results of each of them in a tabular form (Table III; Supplementary Material 1). The results of all the experiments being consistent, he summarized them in another table (Table II; Supplementary Material 1), which led him to conclude about the linearity of dose response with respect to gene mutation (sex-linked lethal) regardless of whether the exposure was acute or split over a long period of time. He also collated the data then available from the literature on radiation-induced mutagenesis and plotted them along with his own data to find dose linearity and mutation frequency (Fig. 4; Supplementary Material 1). In recapitulating Ray-Chaudhuri’s results, our objective is to put the record straight and clarify that much of what Calabrese insinuated was either not relevant or already explained in the paper.

In principle, there was nothing wrong in Calabrese finding points of disagreement with the work presented by Ray-Chaudhuri or Stern, or even with Muller’s conclusions, for that is the essence of science. However, what is unacceptable is the sinister design to denigrate a whole area of science and the scientists with confusing, false, and often irrelevant statements, which he kept repeating with increasing vitriol. It is no less baffling that certain journals kept publishing them with unusual alacrity. It leaves one wondering whether the whole exercise of Calabrese was orchestrated to improve upon a scientific thought or propel ‘Lysenkoism’ in another garb.

## Initial days in Calcutta University and Haldane’s visit

**Figure 2:**
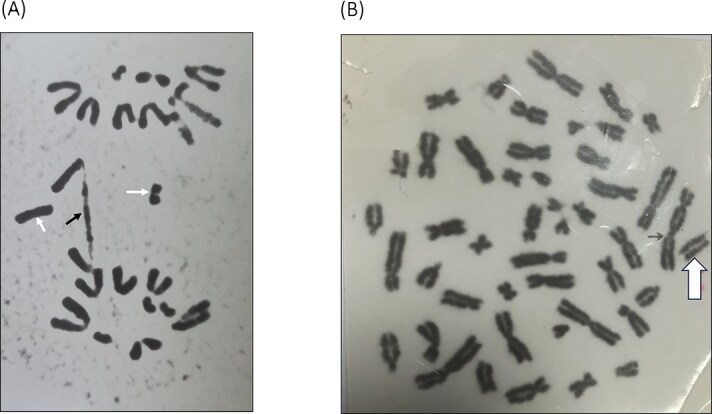
Photographs showing (A) An anaphase I plate of grasshopper chromosomes after X-irradiation showing a dicentric bridge (black arrow) and acentric fragment (white arrows); (B) a X-irradiated metaphase plate from human lymphocyte culture showing a dicentric chromosome (black arrow) with an acentric fragment (white arrow). (Source: Cytogenetics laboratory, BHU).

India was still a British colony when Ray-Chaudhuri returned to live in Calcutta. Since the British were involved in the war, there was severe resource crunch for any research. It is therefore not difficult to imagine that during that time, the research facilities available in Indian laboratories would have been meagre and rudimentary. On his return to India, Ray-Chaudhuri soon realized that the infrastructure required to carry out his genetic studies on fruit flies did not exist, and there was no possibility of creating them in the foreseeable future. More importantly, in the Zoology Departments of those times, there was little appreciation of the kind of work he proposed to do; rather than killing and dissecting animals, he wanted to grow flies for several generations in small milk bottles! Undeterred, Ray-Chaudhuri gainfully utilized his training on chromosome preparation, and he studied radiation-induced aberrations in grasshopper meiotic chromosomes. Though radiation-induced damage in grasshopper chromosomes had been studied earlier by several workers, Ray-Chaudhuri undertook a comprehensive investigation on varied aspects of radiation and chemical-induced clastogenesis under diverse conditions on the semi-aquatic *Gesonula punctifrons*, available all the year-round in Calcutta [7]. He demonstrated that an X-ray-induced dicentric bridge at anaphase was caused by a single hit, and like the point mutations, frequency of the bridges was directly proportional to the dose of radiation but independent of radiation intensity. He carried out a wide variety of experiments on X-ray-induced-chromosome damage, such as the effect of temperature, oxygen deprivation, etc. [8, 9] (Fig. 2A). An important new direction was his initiation of studies on chemical protection against radiation-induced damage in animal chromosomes. These were initial studies constrained by the fact that neither the mechanism of radiation-induced chromosome aberrations nor the nature of potential protectors was clearly understood. On the other hand, with nuclear arsenals expanding in the aftermath of World War II and the bombings of Hiroshima and Nagasaki holocaust, he considered the need to develop such protectors as paramount. He tried various chemicals, especially chelating agents, with mixed results [7]. Despite the limitations, radiation protection remained his major research interest until much later. Besides his passion for radiation cytogenetics, he also studied chromosomes of a variety of insect taxa to unravel their phylogeny and evolution, especially of their sex chromosomes. An important discovery was a ‘neo’ X–Y sex-chromosome system in a species of grasshopper, *Thesiocetrus pulcher* (Grasshoppers are known to lack Y-chromosome with XX female/X male system due to heterochromatinization of a translocated euchromatic autosomal fragment) [10]. During this period, one of the great benefactors of Ray-Chaudhuri was Dr. Subodh Mitra, Director of the Cancer Institute in Calcutta, who made his therapeutic X-ray machine and other facilities available to him for his radiation work. In fact, though never published by him, Ray-Chaudhuri made good chromosome preparations from cancer tissues. However, he counted the normal human chromosome number as 48, as was the prevailing dogma at that time!! Interestingly, as narrated by Ray-Chaudhuri himself, during one of his visits to the cancer Hospital, the first Prime Minister of India, Jawaharlal Nehru, was shown the human chromosomes and introduced to the maker of that preparation with whom he shook hands. Ray-Chaudhuri did not let anyone touch his hand that afternoon until he went home and told his wife of his good fortune that afternoon. A glimpse of Ray-Chaudhuri’s work regimen those days could be had from a little note that his daughter, Ratna Nandi, wrote to one of us (R.R.) recently, ‘When my father was working in Cancer Hospital (CNCI, Chittaranjan National Cancer Institute), I was in high school (*10^th^ standard*) and only remember that he used to spend several nights in the week and the entire Sunday morning there. I guess Dr. Subodh Mitra gave him a lab where he used to work, and Dr. Mitra brought Nehru in that lab to show the chromosome preparations. I vividly remember that incident as he proudly narrated it to us’. In recognition of his fundamental contribution to the area of radiation cytogenetics, Ray-Chaudhuri was elected to the fellowship of the Indian National Science Academy (INSA, then called National Institute of Science) in 1956.

Almost through the decade of 1950, when he served Calcutta University as an Assistant Lecturer, Lecturer, and then a Reader, there were hardly any publications from his lab on *Drosophila* genetics. This is what intrigued the great scholar J.B.S. Haldane, who was one of the adjudicators of Ray-Chaudhuri’s Ph.D. thesis, during his first visit to India in 1951–52. Though highly impressed by his chromosomal work, Haldane was naturally curious to know why he did not pursue with his important genetic studies on *Drosophila*. On being informed of the infrastructural problems in creating and maintaining a *Drosophila* lab, Haldane, in an act of exceptional generosity and farsightedness, actively helped Ray-Chaudhuri in establishing a lab for *Drosophila* Genetics in Calcutta University by writing to Sir S. S. Bhatnagar, the then Director General of Council of Scientific and Industrial Research (CSIR) and advisor to the Prime Minister of India in matters of science policy (Source: Wellcome Trust archives). Thus, the sublime combination of Ray-Chaudhuri’s training and Haldane’s initiative resulted in the birth of the *Drosophila* lab in Calcutta. Now there are more than 150 *Drosophila* labs in India where several hundreds of *Drosophilists* work on a variety of biological questions, not necessarily related to genetics/genomics. Many of these investigators come from the Ray-Chaudhuri lineage. Ray-Chaudhuri initiated his work on *Drosophila* genetics in right earnest at Calcutta, selecting *Drosophila melanogaster* and *Drosophila ananassae* as the species of choice. *D. ananassae* was as cosmopolitan as *D. melanogaster* but was not much explored. His initial work with *D. ananassae* concentrated on gene mapping and male recombination. His association with Haldane and his wife (Helen Spurway) continued unabated during those years. He was also visited by Theodosius Dobzhnasky, one of the most famous evolutionists of his time and the prime proponent of the ‘synthetic theory of evolution’ [11]. Dobzhansky advised him to delve into the Indian *Drosophilid* fauna to explore its taxonomy and use it as a tool to study the mechanism of evolution. However, soon after he moved to Banaras Hindu University (BHU) as Professor and Head of the Department of Zoology. Ray-Chaudhuri’s standardization of aberrations in meiotic chromosomes as a biological dosimeter against radiation and radiomimetic chemicals and the discovery of novel sex chromosome mechanism in insects remained his major research contributions during his tenure at Calcutta University.

## BHU and those transformative years of zoology teaching and research in Indian universities

Banaras Hindu University (BHU) is one of the leading universities and the largest residential university in India. It was established in 1916 near the bank of the river Ganges in the primeval city of Varanasi, the high seat of learning from ancient times. BHU emerged as an offshoot of the struggle for freedom from British rule, an institution to nurture competent and committed youth ready to take on the cause of India’s freedom. It then developed rapidly. After the retirement of its first Head, Prof. A.B. Misra, Ray-Chaudhuri was offered the post of Professor and Head of the BHU Department of Zoology, which was then a small entity in a wing of the Department of Physics with a massive camel skeleton adorning its corridor. However, Ray-Chaudhuri was reluctant to join BHU. He felt that for the time (in 1960), BHU’s zoology department was far too conservative in its curriculum and facilities. Prof. Misra, who was keen on having him, took him to meet the Vice-Chancellor of the University, Justice N.H. Bhagwati, who allocated forthwith funds for the construction of a new, spacious building with modern facilities in keeping with the modernized academic curriculum of Zoology.

The decade of the 1950s was, in many ways, a watershed decade for biology, which catapulted the ‘postmortem-formalin soaked’ biology to the most vibrant discipline capable of addressing central questions related to life itself. The first salvo was fired by Watson and Crick [12], who unravelled the structure of the genetic material, deoxyribonucleic acid (DNA), using X-ray diffraction images. Accessibility of physical and chemical tools and technology to unravel macromolecules not only answered many questions but brought a quantum shift in the ways to examine fundamental biological processes. The human cytogeneticists, Tjio and Levan (1956) confirmed that humans have 46 chromosomes, not 48 as had been believed [13]. By 1959 three well known disorders were discovered to have chromosome aneuploidy [14]. By the end of the 1950’s, biology was at the cusp of a revolution with genetics at the root of it. It is no wonder therefore that Ray-Chaudhuri was so driven to build a new department committed to being an active contributor to the great strides being made in biological sciences.

Ray-Chaudhuri joined BHU in November 1960 along with 3 of his doctoral students and started rebuilding the department with a missionary zeal. As Head of the department, Ray-Chaudhuri appointed new faculty with diverse expertise. He also encouraged those already there to broaden and diversify their academic orbit. More importantly, he placed a major emphasis on research in areas of contemporary life sciences and encouraged colleagues to write research grant proposals and take up projects of biological significance in keeping with the growing trend in global science. Departmental faculty received grants from the Ford Foundation, Population Council, Rockefeller Foundation (all USA), and Nuffield Foundation (UK), besides grants from Indian agencies such as University Grants Commission (UGC), Department of Atomic Energy (DAE), and Council of Scientific & Industrial Research (CSIR). He set up laboratories for Genetics and Cytogenetics with a tissue culture lab for short-term lymphocyte cultures to prepare chromosomes from a wide variety of vertebrates, including humans.

**Figure 3:**
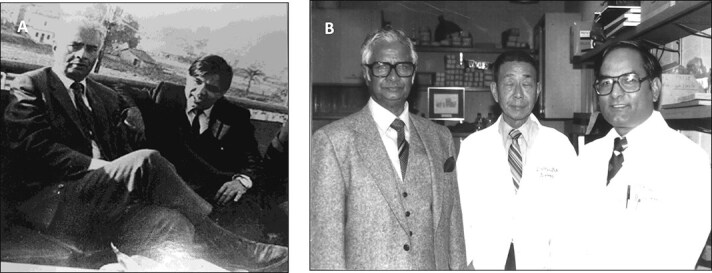
Photographs of S.P. Ray-Chaudhuri with colleagues. (A). Ray-Chaudhuri and Tikaram Sharma at Rajiva Raman's residence in 1975. (B). Ray-Chaudhuri with T.C. Hsu in his lab at MD Anderson Cancer Center, Houston, USA (1980). The third from left is Sen Pathak, who was the first to join Ray-Chaudhuri’s chromosome lab at BHU in 1962 and worked on mammalian (*chiropteran*) chromosomes. He later joined T.C. Hsu’s laboratory and developed as a cancer cytogeneticist, doing phenomenal work on telomeres and cancer. (Photo source: Sen Pathak, Houston, USA).

The Cytogenetics laboratory at BHU was the first to have a tissue culture lab in a University in India. It was set up following a workshop conducted by Harold Klinger (USA) under the aegis of the Indian Council of Medical Research (ICMR) in New Delhi in 1963. One of his research students, Tikaram Sharma, who came with him from Calcutta and later joined the Department at BHU as a faculty member, got training in the workshop and commissioned the tissue culture lab. In this context, Ray-Chaudhuri’s paper on split satellites in the D and G groups of acrocentric chromosomes in lymphocyte cultures was most likely the first paper from India on human chromosomes that was published in an internationally recognized Genetics journal [15]. Tikaram became Ray-Chaudhuri’s main force in building the new department of Zoology both structurally and academically (Fig. 3A).

For Ray-Chaudhuri, the greater challenge was to make fundamental changes in the teaching curriculum. There was strong need to introduce modern subjects and topics so that students understood and enjoyed the new world of biological discoveries. Ray-Chaudhuri had taken upon himself even greater challenge of raising the level of Biology teaching in Indian Universities at large, not an easy task by any means. In his address as the First President of the All India Society of Cell Biology in 1977, he alluded to some of these difficulties in these words ‘To foster teaching and research in cell biology in my mind are the most important task confronting us…The only remedy is to have graded course in the molecular aspects of cell biology from B.Sc. onwards for the simple reason that the cell is the common denominator of all the living forms. We should realize that cell biology cannot remain at the periphery of biology course as an esoteric subject but must come at the center, and around it the whole course should be built up.’ Ray-Chaudhuri retired from BHU in 1971 at the age of 63. His services were actually extended by 3 years to help him refine the teaching edifice that he had begun to build. Before retirement, he succeeded in getting the ‘Special Assistance Program’ of UGC of India to BHU Department of Zoology, which was later elevated to the level of the’ Centre of Advanced Studies’, a distinction that the department still enjoys. The “Ray-Chaudhuri era” at BHU is unarguably the finest period of the departmental history. Leading human cytogeneticist, T. C. Hsu, MD Anderson Cancer Center, Houston, Texas, while writing foreword to the book, ‘Trends in Chromosome Research’ brought out in Ray-Chaudhuri’s honour on his 70^th^ birthday, succinctly described him as a ‘Scholastic Builder’ [16] (Fig. 3B).

## Research contribution at BHU

Administrative duties and the mission to achieve his objective consumed much of his time from research, but Ray-Chaudhuri was as resolute about his research as he was for bolstering the teaching standards. That amalgam of a teacher and a researcher worked well for him. He was clear from the beginning that rather than focusing on one single area, he would produce students working in different areas of genetics/cytogenetics so that the subject developed in the country. His commitment to expand in areas of *Drosophila* taxonomy and evolution, cytogenetics of diverse vertebrates, besides radiation genetics and cytogenetics led him to diversify his research activities. The objective was to produce competent researchers capable of foraying in different areas of biology using diverse model systems to answer novel questions and contribute significantly to the area they chose. Hence, even as a researcher, he remained quintessentially a teacher.

Ray-Chaudhuri’s research interest was undoubtedly in radiation-induced mutagenesis and chromosome damage. The tissue culture lab set up by BHU in 1962 was created primarily to prepare human chromosomes from phytohaemagglutinin (PHA)-stimulated human lymphocyte cultures for 48–72 hours that could be easily irradiated and grown post irradiation. Ray-Chaudhuri turned his attention to human chromosome damage by X-rays using dicentric chromosomes and chromatid exchanges as robust markers of genetic damage in pre-replicative and post-replicative phases of cell cycle, respectively. A concerted effort by his lab and those of Mike Bender (USA) and H. John Evans (UK) revealed the difficulty in drawing consistent dose–response relationships, probably due to proliferative heterogeneity of the PHA-stimulated lymphocytes which was not so obvious at that time (Fig. 2B). Nearly 2 decades later, the International Atomic Energy Agency (IAEA), took up chromosomal dosimetry as an international project which was led by RN Banerjee (IAEA, Vienna) and AT Natarajan (Leiden). Tikaram Sharma of the Cytogenetics Laboratory of BHU was the lone participant in this project from India [17, 18]. Ray-Chaudhuri also doggedly pursued his efforts to identify chemical radioprotectors against radiation damage [19]. Once it became possible for him to work on *Drosophila*, he devised ways to restrict radiation-induced point mutations and chromosome damage. Reduced glutathione was found to be protective against point mutations and chromosome damage [20]. Even after his retirement, the Cytogenetics and Genetics labs at BHU continued using diverse model systems, viz, the Indian Muntjac, *Muntiacus muntjak*, which is the mammal with the lowest chromosome numbers (6 female/7 male) and the flying squirrel, *Hylopetes alboniger*, which has a large amount of heterochromatin in autosomes. The muntjac was found to exhibit the sex-specific differential radiosensitivity [21]. One of us (A.N.J.), who worked in the same lab for a Ph.D, reported enhanced radiosensitivity in workers occupationally exposed to low levels of diagnostic X-rays, a result important both to researchers and policy makers in India [22]. With the advent of molecular biology and recombinant DNA technology, attention was naturally shifted to DNA damage and repair in germ cells and in cancers [23].

**Figure 4:**
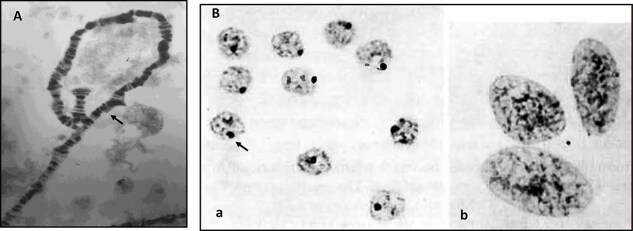
Photomicrographs (A). Part of the polytene chromosomes of *D. ananassae* showing an inversion (α inversion, see arrow) in the chromosome arm 2 l (source genetics laboratory, BHU) and (B) W-chromatin in interphase nuclei of female cells. (a): Interphase nuclei from somatic cells of the snake *Bungarus caeruleus* female showing W-chromatin (arrow) in all the nuclei in contrast to (b) interphase nuclei of the male *B. caeruleus* which lack this chromatin (Ray-Chaudhuri *et al.* 1970; reproduced with permission from S. Karger).

Prompted by Dobzhansky’s comment about lack of research in the fields of evolution as well as taxonomy, Ray-Chaudhuri extended his work in areas of population genetics using population cage experiments as well as inversion polymorphism in polytene chromosomes in diverse populations of *D. ananassae* (Fig. 4A) as the vehicle of evolutionary divergence [24]. Also, the cytogenetic map of the polytene chromosome of *D. ananassae* prepared by Ray-Chaudhuri and Jha [25] is universally used as the standard map for this species. His students, B.N. Singh and A.P. Jha, extended work on evolutionary genetics in a big way using *D. ananassae* and some other indigenous species for the study. In contrast to the fact that the males in *Drosophila* do not undergo crossing over during meiosis, Ray-Chaudhuri and Kale [26] showed that in *D. ananassae*, not only that crossing over occurs in males but that it is of meiotic origin [27]. Kale also showed radiation-induced crossing over in males of *D. melanogaster*. Ray-Chaudhuri also initiated studies on *Drosophila* taxonomy with one of his students, J.P. Gupta, who discovered several new species and reported many for the first time from the Indian subcontinent [28], and went on to become an authority on South Asian *Drosophilids*.

Ray-Chaudhuri used short-term lymphocyte cultures and bone marrow (*in vivo*) for chromosome preparation across the vertebrate taxa and cultivated a group of highly motivated and accomplished students who exhaustively explored the vertebrate fauna, particularly reptilian, avian, and mammalian species, elucidating their karyotypes and chromosomal evolution. The chromosome analysis of Indian birds showed the macro- and micro-chromosomes and also the integrity and permanence of the latter through meiotic cell division [29]. Similar studies in reptiles led to elucidation of the origin and evolution of sex chromosomes in snakes, showing that whereas phylogenetically less evolved snakes (e.g. nonpoisonous rat snake, John’s snake) lacked sex chromosomes, the more evolved ones (e.g. krait, cobra, viper) had highly differentiated sex-chromosomes which, harbored female heterogamety (ZW chromosomes in female/ZZ males) like birds and unlike mammals. It was Ray-Chaudhuri’s pioneering work [30, 31] that provided incontrovertible evidence that the differentiation of the sex chromosomes from a pair of homologous chromosomes occurred when one of a pair of homologues got heterochromatinised by the accumulation of repetitive DNA and reduction in size forming the W-chromosome in females which formed the ‘W-chromatin’ in female soma (Fig. 4B). Later, his student, Lalji Singh, while working with Ken Jones in the Institute of Animal Genetics, Edinburgh, isolated a W-chromosome-specific satellite DNA, Bkm, from the DNA of the banded krait, *Bungarus fasciatus* and the viper, *Elaphe radiate* [32]. Bkm comprised repeats of GATA sequences that were later used by Singh to perform DNA fingerprinting that became legally acceptable as evidence in the Indian legal system. After retiring from active service at BHU, Ray-Chaudhuri went back to Calcutta and remained active with his research as Scientist Emeritus of the Indian Council of Agriculture Research (ICAR) (1972–75) and continued to publish research until much after his retirement [33]. Indian National Science Academy (INSA) honoured him with the S.L. Hora Award for his pioneering contribution to Biological Sciences in 1986.

Though most of his research work was published in reputed foreign journals, he was a strong advocate for developing strong research journals from India. He encouraged Indian scientists to publish some of their research in those journals, as mentioned earlier by one of us [34]. He believed that good research journals are essential to the development of science in the country, for it promotes a high level of discourse among scientists and flags Indian science internationally. Even though there were relatively few journals published in India at that time and some did not merit sufficiently high standard, he published some of his work in them [35, 36, 37]. Indian science was thereby the beneficiary of this perceptiveness and dedication. People like Ray-Chaudhuri were among those who took the task of elevating India through education and scientific temper. T.C. Hsu described him as a scholastic builder [12]. Such ‘builders’ are never too many and are always in demand in any and every society.

## Supplementary Material

Suppl_Material_1_Ray-Chaudhuri_Bunsen_Roscoe_Law_260326_geag016

## Data Availability

The written permission has been obtained from the Royal Society of Edinburgh to use this paper as Suppl. Material 1. This has been acknowledged in the acknowledgment section.
