# Supplementary material for: Sachi Prasad Ray-Chaudhuri: Drosophila genetics and mutagenesis in Indian science
Source: Mutagenesis. 2026 Apr 1;41(4):167–74. doi: 10.1093/mutage/geag016 (PMC13310013; doi:10.1093/mutage/geag016)
Supplement: Suppl_Material_1_Ray-Chaudhuri_Bunsen_Roscoe_Law_260326_geag016 [file suppl_material_1_ray-chaudhuri_bunsen_roscoe_law_260326_geag016.pdf]

IX.—The Validity of the Bunsen-Roscoe Law in the Production of Mutations by Radiation of Extremely Low Intensity. By S. P. Ray-Chaudhuri.

Communicated by Dr A. W. Greenwood, Institute of Animal Genetics, University of Edinburgh. (With Five Text-figures.)

(MS. received May 31, 1944. Read July 3, 1944)

Introduction

The Bunsen-Roscoe law states that the effect of radiation is always the same regardless of the variation in the intensity or in the time of radiation as long as the product of the two is kept constant. This law holds true for many ordinary physico-chemical reactions over a wide range of intensities. The present investigation was undertaken to test the validity of the Bunsen-Roscoe law by studying the frequency of (1) sex-linked lethal mutations and (2) translocations in *Drosophila melanogaster* at much lower intensities of radiation than those previously tried.

Method

Inseminated females of *Drosophila melanogaster* were treated with gamma-rays of radium for 720 hours (30 days) continuously. There are two serious experimental difficulties in treating inseminated females. It is essential to have a large number of progeny for a statistically significant result because of the low number of mutations expected at a low dosage. Flies, after a month's treatment, are comparatively old and their output of eggs decreases very considerably. The other difficulty arises from the fact that spermatozoa under the usual conditions of temperature and food are not retained in the body of the females but are used up in the process of egg-laying. In order to overcome these difficulties a test experiment was carried out and the data obtained are summarised in Table I.

Table I.—Retention of Sperms in Inseminated Females of Wild Samarkand *D. melanogaster*

Groups	No. of Flies tested after 720 Hours	No. of Eggs Laid	No. of Larvæ	Per cent. of Fertility	Reproduction Rate per Fly
A	13	3186	1908	59·5	146·7
B	20	2271	1309	57·6	65·4
C	19	725	201	27·2	10·5
D	14	486	53	10·9	3·7

The conditions under which the flies of the different groups were kept are as follows:—

A: inseminated flies were kept on syrup food (D.I.S. 6) at 8° C.
B: ,, ,, ,, ,, ,, at room temperature.
C: ,, ,, ,, on Offermann's yeast food (D.I.S. 6) at 8° C.
D: ,, ,, ,, X-rayed with *ca.* 1500 r. and then kept on syrup at 8° C.

The performance of flies of group A being the best, the same method was adopted for the experiments.

With a view to detecting sex-linked lethals and translocations at the same time, *Cy*; D ♂♂ were used to inseminate C*l*B ♀♀. The latter were treated for 720 hours while on syrup food at 8° C. before allowing them to breed. F_1 females of the constitution $\frac{ClB\ +\ +}{+\ Cy\ D}$ were mated to

"*scar*" males. However, during the experiment it was found impracticable to use the same F_1 flies for detecting both lethals and translocations because of the difficulty to obtain the requisite numbers of F_1 females of the right kind. It was, therefore, decided to use ♀♀ of a homozygous stock of *sc v Δ 49 f* instead of C*l*B, which were crossed to $sc^8\ w^a$ B ♂♂. This mating gave a large number of F_1 ♀♀ which could be used for testing the presence of sex-linked lethals.

Homozygous *bw*; *e*; *ey* ♀♀ were crossed to Samarkand males for detecting the translocations. The F_1 males were backcrossed individually to *bw*; *e*; *ey* ♀♀ and the distribution of the markers was followed in each of the F_2 families.

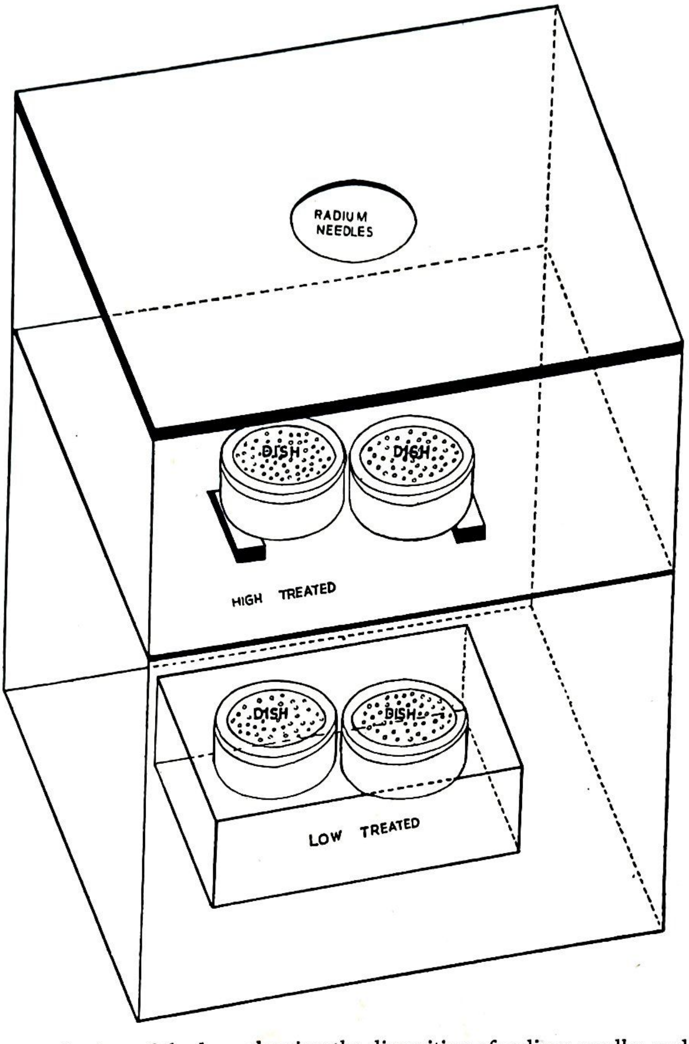

FIG. 1.—Isometric view of the box, showing the disposition of radium needles and dishes.

The exposures to radiation were carried out in a wooden box placed in a Hearson's patent refrigerator, which was kept running at 8° C. with an occasional rise of 2° to 3° for brief periods. A similar box containing the controls was kept in another refrigerator of the same type. Fig. 1 is an isometric view of the wooden box in which the flies were kept during the radiation, and fig. 2 is a sectional elevation. Seventy mg. of radium were distributed in 54 platinum needles which were arranged in a single layer in a dish-shaped depression cut into the centre of the top of the box, giving in effect a uniform distribution of radium over a circular arc of 5 cm. diameter. Fig. 3 shows this arrangement and also shows one of the dishes in which the flies were irradiated. The round bakelite dishes were provided with transparent lids of celluloid *ca.* 1 mm. thick and having a few perforations. Each contained a layer of food 1·3 cm. thick on which the flies were spread out practically evenly over a circular surface of 7·5 cm. in diameter.

Experiments and Results

(A) *Sex-linked Lethals*

Three intensities of radiation were employed, obtained by varying the distance between the radium and the flies. As indicated in fig. 2, two dishes were exposed at a time for the two lower intensities; the distances (measured from centre of radium to centre of the layer of flies)

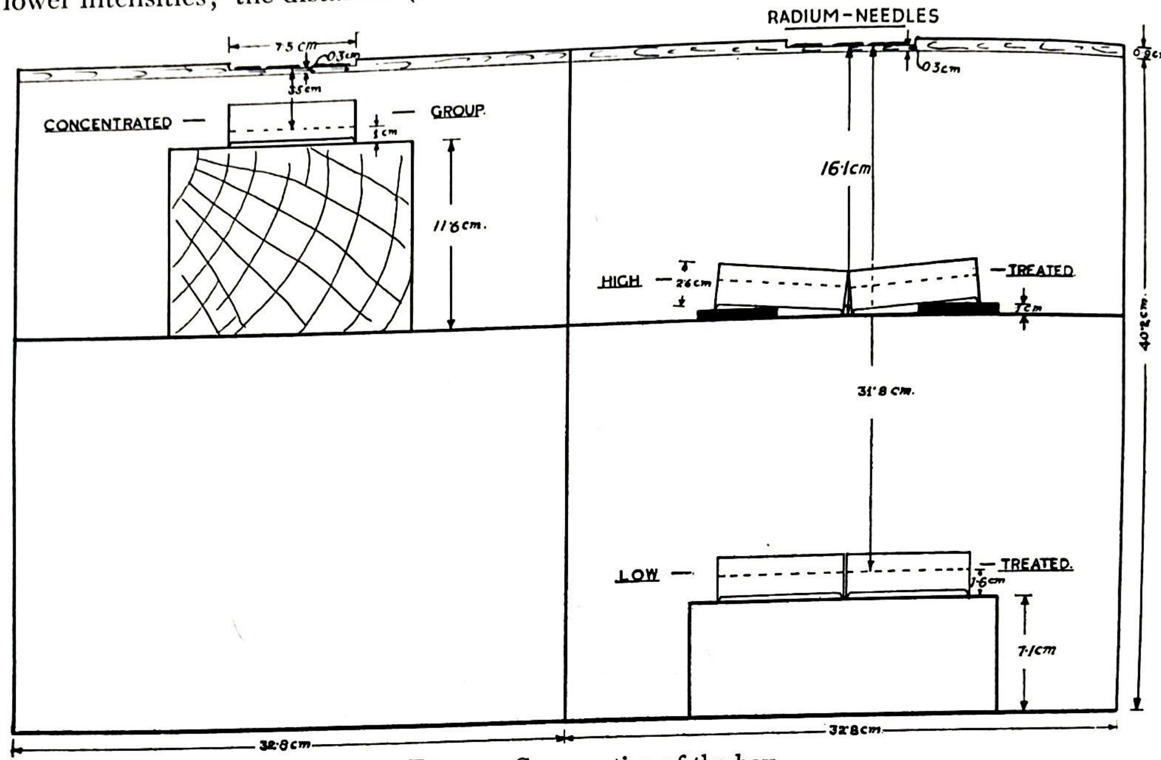

Fig. 2.—Cross-section of the box.

being 31·8 and 14·2 cm. respectively. In the high intensity experiment the distance, centre to centre, was 3·5 cm.

The dose-rates were calculated, assuming that with the filtration employed 1 milligramme of radium at 1 cm. gives a dose-rate of 8 roentgen per hour. The dose-rate listed in Table II

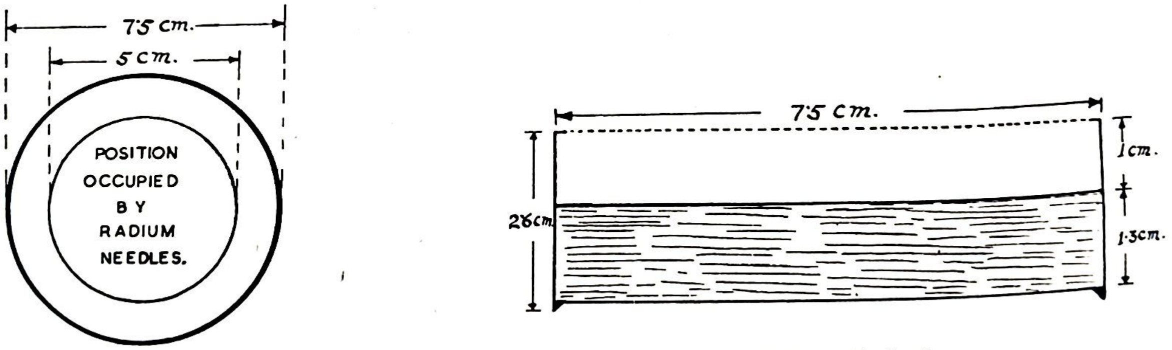

Fig. 3.—Disposition of radium needles, section of dish with food.

for the two lowest intensities are calculated in this way by the inverse square law for distance between flies and radium of 31·8 and 14·2 cm. respectively. If the dose-rate at the 3·5 cm. distance is calculated in the same way it will be underestimated, since 3·5 cm. is the minimum distance between flies and radium, and the extreme distance is considerably greater. Formulæ are available (Mayneord, 1932) for calculating the dose-rate at any point due to radium distribution in a circular layer, and these formulæ were used to calculate the average dose-rate received by the flies at the 3·5 cm. distance, which actually is the dose-rate, which can be produced by a *concentrated* source of 70 mgm. at a distance of 4·4 cm. The experimental results are summarised in Table II.

TABLE II.—FREQUENCY OF SEX-LINKED LETHALS PRODUCED WITH VARIOUS INTENSITIES OF γ-RADIATION

Distance in cm.	Dose-rate r. per Hour	Duration of Exposure in Hours	Dose (Roentgen)	Per cent. of Lethals	Per cent. of Lethals due to Treatment	Per cent. of Lethals induced per 1000 r.
Control	..	..	..	0·317 ± 0·095	..	..
31·8	0·556	720	400	1·53 ± 0·20	1·21 ± 0·22	3·03 ± 0·55
14·2	2·78	720	2000	6·675 ± 0·85	6·36 ± 0·85	3·18 ± 0·42
3·5	29·0	45	1300	5·25 ± 0·82	4·93 ± 0·83	3·79 ± 0·64

Taking into account the magnitude of the statistical error, these results do not indicate any significant dependence of yield upon dose-rate. The data were obtained from eight series of experiments and are detailed in Table III. In all the control series added together 3471 fertile cultures were examined and 11 lethal mutations were obtained, giving the percentage of 0·317 ± 0·095. The variation in numbers between different control series was not significant, as even the total number of spontaneous sex-linked lethals is quite low although it has a fairly high standard error. If we compare this figure with data obtained by different workers, we find the present value to be a little higher than that most frequently obtained, yet not higher than values obtained quite commonly (*cf.* Demerec, 1937). The control value in the present experiment is very important because of the low number of mutations expected in the low treated groups. Beginning with series VI flies of the stock, sc^8 B w^a were used as controls; they gave the same mutation rate as the previously used Samarkand flies.

TABLE III.—FREQUENCY OF SEX-LINKED LETHALS IN CONTROL AND TREATED SERIES

Series	Control		Low Treated (31·8 cm. distance) 400 r.		High Treated (14·2 cm. distance) 2000 r.	
	No. of F_2 Cultures Tested	No. of Lethals	No. of F_2 Cultures Tested	No. of Lethals	No. of F_2 Cultures Tested	No. of Lethals
I . . .	520	2	510	9	102	7
II . .	435	2	436	9	80	7
III . .	604	3	525	8	122	10
IV * . .	..	..	593	9	121	4
V . .	537	2	543	7	121	10
VI . .	432	1	379	4	99	6
VII . .	632	1	457	6	96	7
VIII . .	311	..	412	7	127	7
Total . .	3471	11	3855	59	868	58
Per cent. of Lethals	0·317 ± 0·095		1·53 ± 0·1995		6·675±0·846	

The data obtained in the low- and high-treated series agree well with the conception of a direct proportionality between dosage and mutation rate, independent of the intensity, even with an intensity as low as 1/100 r. per minute. The result also compares favourably with that obtained in the concentrated series when the distance was reduced to 3·5 cm. and the duration of exposure to 45 hours.

The results of the present experiment, together with the published results of other works at higher intensities, are shown in fig. 4. The graph is obtained by plotting the frequency of mutations per 100 r. against the intensity of radiation and shows that the frequency for a given dose is constant, regardless of intensity. Thus it is concluded that the Bunsen-Roscoe law

* The control series No. IV. has failed owing to an accidental drop of temperature in the refrigerator.

holds in the production of mutations by radiation within the very wide limits of intensity of 0·01 r./min. and 300 r./min. (Timofeeff-Ressovsky and Zimmer, 1935), a ratio of 1 to 30,000. The time factor varied from 5 min. to 720 hours, a ratio of 1 to 8640.

(B) *Translocations*

Three series of experiments were carried out, they correspond to the last three series (VI, VII, and VIII) of the sex-linked lethals. Total doses were 2000 and 400 r. respectively, and the flies were exposed to radiation for 720 hours. The summarised data of these series are

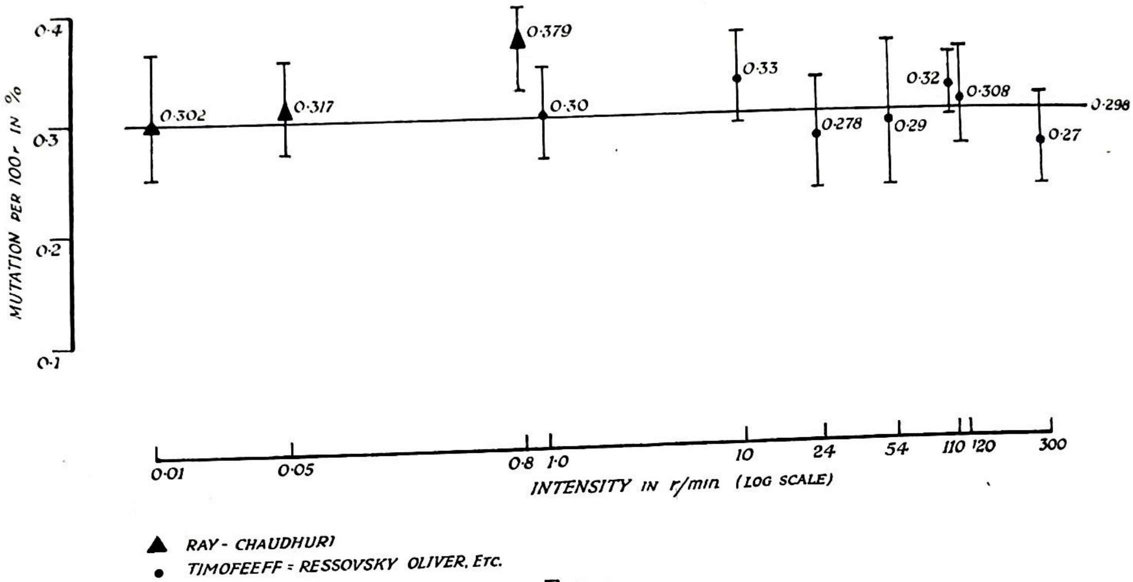

FIG. 4.

compared with those obtained in the "concentrated" group in which 1300 r. and 260 r. were given in 45 and 9 hours respectively. The data are given in Table IV.

TABLE IV.—FREQUENCY OF TRANSLOCATIONS PRODUCED WITH VARIOUS INTENSITIES OF γ-RADIATION

Distance in cm.	Dose-rate r./Hour	Duration of Exposure in Hours	Dose (Roentgen)	Dose ²	No. of F_1 Males Tested	No. of Trans-locations Obtained	Per cent. of Trans-locations
31·8	0·556	720	400	$0·16 \times 10^6$	1208	1	0·08 ± 0·08
14·2	2·78	720	2000	$4·00 \times 10^6$	469	21	4·47 ± 0·96
3·5	29·0	45	1300	$1·69 \times 10^6$	473	19	4·01 ± 0·90
3·5	29·0	9	260	$0·07 \times 10^6$	601	1	0·17 ± 0·17

The difference between the frequencies of translocations at the dosage of 2000 and 1300 r. at the two intensities is 0·46 ± 1·32 per cent.; for 400 and 260 r. the difference is 0·09 ± 1·8 per cent. The data of Table IV are shown graphically in fig. 5 plotted against $(\text{Dose})^2$. The points for the higher intensity (29·0 r./hour) may or may not fall into a different line from the points for low intensity (0·556 r./hour). In fig. 5 a point for X-rays, dose 1500 r. obtained by Makhijani (unpublished), is also inserted. He observed 2·57 ± 0·3 per cent. translocations after 1500 r., but since he scored only translocations between chromosomes II and III his figure is multiplied by 42/38, since in our present experiments we scored 42 translocations altogether, of which 38 were between chromosomes II and III; with this correction the point at 2·85 ± 0·3 is obtained. Taking Makhijani's data into consideration, it appears unlikely that any variation of yield with dose-rate actually occurs. The observed

difference, if it is real, is in the direction of the lower intensity being less effective than the higher intensity.

Discussion

The time distribution of ionisations in the irradiated sperm was found to be as follows:—

(A) Low treated, distance 31·8 cm.: 1·1 ion pairs per sperm per hour.
(B) High treated, ,, 14·2 cm.: 5·5 ,, ,, ,, ,,
(C) Concentrated, ,, 3·5 cm.: 58·0 ,, ,, ,, ,,

The data are obtained by taking into account the fact that 1 r. unit gives 2×10^{12} ion pairs per g. of organic substance, and that the volume of the Drosophila sperm head is 1×10^{-12} cm.^{3}. From these results it is reasonable to conclude that individual ionisations, acting within a rather

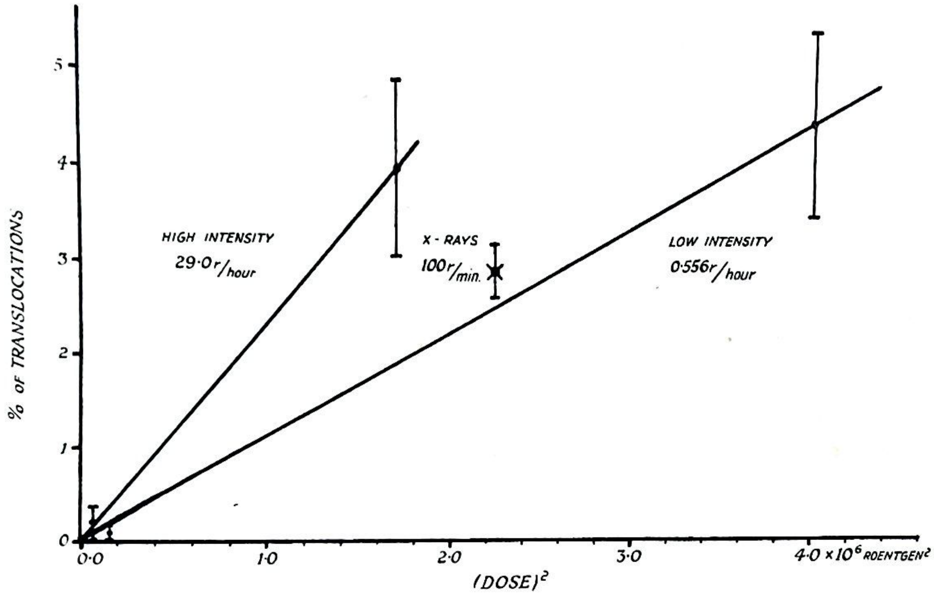

Fig. 5.

limited space, cause the necessary changes for the production of mutation. Neither the distribution of ions in space within wide limits, or in time, has any differential effect on the mutation process. For an individual ion, no matter whether or not it is associated with other ions, there is a certain definite chance of a mutation being produced. These minute definite chances summate exactly in time to produce the observed frequency.

Three factors might alter the validity of the Bunsen-Roscoe law in relation to mutations: (*a*) a restitution process affected by ion concentration; (*b*) change in radio-sensitivity during the time of treatment; (*c*) the existence of a threshold intensity, owing to the action of more than one ion being required in a given space and time. Data obtained by Muller (1928) and Harris (1929) rule out the possibility of a back-reaction. While Timofeeff-Ressovsky (1931) found no evidence of change in radio-sensitivity by aging the sperm of *Drosophila*, Offerman (1939) reported the finding of a higher mutation rate in older sperm. The discrepancy between their data may be explained by assuming the renewal of sperms in the testes during ageing. In order to avoid this possibility in our experiments inseminated females were used. No significant difference of mutation rate was observed in the two groups, one treated for 720 hours; the other for 45 and 9 hours only. Thus our results contradict the existence of a threshold intensity. It is very improbable that one ion pair, while unable to produce mutation, could nevertheless have its effect stored up for hours and then so interact with the effects of the later ion pairs to give a frequency of mutations exactly proportional to the total number of ion pairs. On the basis of these considerations, and of the present data, the applicability of the Bunsen-Roscoe law to the mutation reaction is held to be valid.

Our data also show that irrespective of the mechanism of the production of chromosome rearrangements, these are produced in the spermatozoa independently of the time distribution

of the ionisation, and also independently of such differences in space distribution as characterise X- as compared with gamma-rays. The frequencies of translocations obtained in the two groups, one treated for 720 hours, the other for 45 hours, indicate that those chromosome breaks which were produced during the earlier portion of the month-long treatment remained open; union of broken chromosomes (restitution or new rearrangement) occurs only after fertilisation.

Summary

1. Sperms of *Drosophila* were irradiated in the body of impregnated females with 70 mgm. of radium for 720 hours at two dosages of 2000 r. and 400 r., with an intensity of 0·556 r. and 2·78 r. per hour respectively.

2. The frequency of sex-linked lethals produced was found to be directly proportional to the dosage and independent of the intensity of irradiation.

3. The mutation rate was found to be independent also of the duration of the treatment within the range from 5 minutes to 720 hours.

4. The data are not sufficient to decide whether the frequency of translocations follows the Bunsen-Roscoe law.

Acknowledgments

The author wishes to express his indebtedness to Professor H. J. Muller for his advice and guidance throughout the course of his investigation. He is greatly indebted to Dr D. E. Lea, Strangeways Laboratory, Cambridge, for his criticism and help in preparing the MS. for publication, and to Professor W. V. Mayneord, Royal Cancer Hospital, London, for his suggestions. Thanks are due to Dr E. Murison, Royal Infirmary, Edinburgh, for supervising the set-up of the experiments. The loan of 70 mgm. of radium from the British Empire Cancer Campaign is gratefully acknowledged. The experiments were aided financially by the Carnegie Trust.

REFERENCES TO LITERATURE

Demerec, M., 1937. "Frequency of spontaneous mutations in certain stocks of *Drosophila melanogaster*," *Genetics*, xxii, 469–478.

Harris, B. B., 1929. "The effects of aging X-rayed males upon mutation frequency in *Drosophila*," *Journ. Hered.*, xx, 299–302.

Mayneord, W. V., 1932. "The distribution of radiation around simple radio-active sources," *Brit. Journ. Rad.*, v, 677–715.

Muller, H. J., 1928. "The problem of genic modification," *Zeits. Indukt. Absramm. u Vererbunsl.*, Supl. 1, 234–260.

Offermann, C. A., 1939. "Effect of aging on the frequency of induced mutations," *Genetics*, xiv, 81–82.

Oliver, C. P., 1932. "An analysis of the effect of varying the duration of X-ray treatment upon the frequency of mutations," *Zeits. ind. Asst. Veverb. Lehre*, lxi, 447–488.

Timofeeff-Ressovsky, N. W., 1931. "Einige Versuche an *Drosophila melanogaster* über die Art der Wirkung der Röntgenstrahlen auf den Mutationsprozess," *Roux' Arch. Entwicklungsmech.* cxxiv, 654–665.

Timofeeff-Ressovsky, N. W., and Zimmer, K. G., 1935. "Strahlengenetische Zeitfaktorversuche an *Drosophila melanogaster*," *Strahlentherapie*, liii, 134–138.

(*Issued separately November* 22, 1944)
